# Mitochondrial mass and mitochondrial membrane potential of peripheral lymphocytes: promising biomarkers of systemic lupus erythematosus

**DOI:** 10.3389/fmolb.2025.1585847

**Published:** 2025-06-06

**Authors:** Guanfei Zhao, Haolong Li, Yutong Miao, Linlin Cheng, Yuying Chen, Yuan Huang, Hongyun Zhao, Yongmei Liu, Yipei Jing, Shasha Wang, Yongzhe Li, Rui Zhang

**Affiliations:** ^1^ Department of Clinical Laboratory, Beijing Chao-Yang Hospital, Capital Medical University, Beijing, China; ^2^ Department of Clinical Laboratory, State key Laboratory of Complex, Severe and Rare Diseases, Peking Union Medical College Hospital, Chinese Academy of Medical Science and Peking Union Medical College, Beijing, China

**Keywords:** systemic lupus erythematosus, mitochondrial mass, mitochondrial membrane potential, mitochondrial DNA, lymphocytes

## Abstract

**Background:**

Mitochondrial dysfunction is implicated in the pathogenesis of systemic lupus erythematosus (SLE). Single-cell mitochondrial mass (SCMM), low mitochondrial membrane potential (MMP-Low) in lymphocytes, and circulating mitochondrial DNA (mtDNA) can reflect mitochondrial impairment and may serve as potential novel biomarkers for SLE.

**Purpose:**

We investigated the diagnostic utility of MMP-Low and SCMM in lymphocytes, as well as circulating mtDNA levels, in patients with SLE and examined their correlation with disease activity.

**Methods:**

Flow cytometry was performed to detect MMP-Low and SCMM in peripheral lymphocytes from patients with SLE (n = 52) and healthy controls (HCs, n = 30). The level of circulating mtDNA was quantified using PCR.

**Results:**

Patients with SLE exhibited significantly decreased MMP-Low in some peripheral lymphocyte subsets. Meanwhile, significantly increased SCMM in some lymphocyte subsets and circulating mtDNA were observed in patients with SLE. CD8^+^ T naïve (Tn) cell MMP-Low, CD8^+^ T effector memory cell MMP-Low, CD8^+^ T central memory (Tcm) cell MMP-Low, and SCMM-CD8^+^ Tn cells demonstrated a moderate diagnostic value for SLE, with an area under the curve (AUC) above 0.8. Both CD4^+^ Tcm MMP-Low and SCMM-CD3^+^CD4^+^ T cells were significantly associated with the SLE Disease Activity Index 2000 (SLEDAI-2K) and circulating mtDNA levels. These markers also showed significant alternations between inactive and active SLE.

**Conclusion:**

Our data showed that patients with SLE exhibit mitochondrial dysfunction. Several MMP-Low and SCMM in CD8+T cell subsets could serve as potential biomarkers for diagnosing SLE. Additionally, CD4^+^ Tcm MMP-Low and SCMM-CD3^+^CD4^+^ T cells were associated with SLE disease activity.

## Background

Systemic lupus erythematosus (SLE), a prototypical autoimmune disease, is characterized by multiple organ involvement and varied clinical manifestations (Lazar and Kahlenberg, 2023; Caielli et al., 2023). It is more common in young female individuals. Due to the heterogeneity of the disease, early diagnosis of SLE still remains challenging (Dörner and Furie, 2019). Furthermore, the pathogenesis of SLE is complex. Numerous studies have revealed that the loss of immune tolerance to self-nucleic acids and self-antigens, immune cell hyperactivation, activation of the interferon system, and defects in apoptotic cell clearance contribute to SLE pathology (Owen et al., 2022). In addition, mitochondria also play an important role in SLE pathogenesis through various mechanisms (Caielli et al., 2023).

Mitochondria, critical organelles, play a key role in supplying energy for multiple cell activities and regulate cell activation and differentiation by generating adenosine triphosphate (ATP) through oxidative phosphorylation (Mills et al., 2017). Mitochondrial dysfunction contributes to SLE pathogenesis through excessive mitochondrial reactive oxygen species (ROS) production, mitochondrial DNA (mtDNA) damage, and impaired mitophagy (Zhao et al., 2022). In addition, mitochondrial components have also been proven to be the source of extracellular and intracellular danger-associated molecular patterns in SLE, leading to abnormal inflammatory response and immune cell activation (Caielli et al., 2023). Moreover, oxidative stress-mediated damage, including lipid peroxidation and protein modification, has been implicated in SLE. Specifically, 4-hydroxynonenal (HNE)-modified proteins contribute to autoimmune responses and serve as biomarkers of oxidative stress in lupus patients (Khan et al., 2016). Mitochondrial mass (MM) is a hallmark of mitochondrial fitness, and increased MM can induce abnormal activation of immune cells (Yu et al., 2020; Callender et al., 2020). Furthermore, impaired mitochondrial quality control has also been observed in SLE, and this change causes the dysfunction of immune cells in SLE (Caielli et al., 2023). However, the MM of immune cells in patients with SLE still remains unclear.

In this study, a new immunofluorescence technology was used to detect single-cell mitochondrial mass (SCMM) and low mitochondrial membrane potential (MMP-Low) in immune cells from patients with SLE (Puleston, 2015; Author Anonymous, 2021). First, the distribution of immune cells, including CD3^+^ T cells, B cells, NK cells, CD4^+^ T cells, CD8^+^ T cells, CD4^+^ T naïve (Tn) cells, CD4^+^ T effector (Tef) cells, CD4^+^ T central memory (Tcm) cells, CD4^+^ T effector memory (Tem) cells, CD8^+^ Tn cells, CD8^+^ Tef cells, CD8^+^ Tcm cells, and CD8^+^ Tem cells, was analyzed. Furthermore, flow cytometry with Mito dye staining was used to detect the SCMM of peripheral immune cells. SCMM could more sensitively reflect the processes involved in mitochondrial quality control in immune cells, including mitophagy, mitochondrial dynamics, and mitochondrial biogenesis (Miwa et al., 2022; He et al., 2023; Pang et al., 2022). In addition, we analyzed the relationship between SCMM and MMP-Low in immune cells and mtDNA levels in patients with SLE. This analysis aimed to evaluate the diagnostic utility of MMP-Low and SCMM in lymphocytes, as well as circulating mtDNA levels, and examine their correlation with SLE disease activity.

## Methods

### Study participants

A total of 52 patients with SLE and 30 age- and sex-matched healthy controls (HCs) were enrolled between May 2022 and July 2022 at Peking Union Medical College Hospital (PUMCH). All patients with SLE fulfilled the 1997 classification criteria of the American College of Rheumatology (ACR) (Hochberg, 1997); individuals with other autoimmune diseases, infections, pregnancy, and malignant tumors were excluded from this study. Systemic Lupus Erythematosus Disease Activity Index 2000 (SLEDAI-2K) was used to evaluate the disease activity of patients with SLE (Gladman et al., 2002). Patients with SLE whose SLEDAI-2K < 6 were regarded as inactive SLE, and those whose SLEDAI-2K ≥ 6 were regarded as active SLE. All of the HCs had a normal range in liver and kidney function tests and blood and urine routine tests, and they did not have any autoimmune diseases, infections, malignant tumors, pregnancy, or irrelevant chronic diseases. This cross-sectional study was approved by the Research Ethics Commission of PUMCH (JS-2156). Informed written consent was waived for the remaining routine test samples.

### Blood sample collection and processing

Whole blood was collected from each individual in ethylenediaminetetraacetic acid (EDTA)-treated tubes. After receiving the sample, 200 μL of fresh whole blood was taken from an EDTA-treated tube for flow cytometric analysis. The remaining whole blood was centrifuged at 3,000 × g for 10 min, and the plasma was collected into RNase/DNase-free Eppendorf tubes (MCT-150-C, Axygen, Wujiang, China). Plasma samples were then stored at −80°C for DNA analysis until mtDNA copy number was evaluated.

### Flow cytometric analysis

Each whole blood sample was divided was added to two corresponding flow tubes (100 μL whole blood/tube), each containing flow antibodies. The tubes were gently mixed and incubated in the dark for 15 min at room temperature. Then, 2 mL of lysing solution (NH Lysis Solution, 10×) was added to the tubes, mixed again, and incubated in the dark for an additional 15 min at room temperature. The residual lysed specimens were removed following centrifugation at 300 *g* for a duration of 5 min. One flow antibody panel including PE-CD3, PE-CD56, FITC-CD8, FITC-CD19, and PE-CY7-CD4 (UBBIO, Zhejiang, China) was applied to detect CD3^+^ T cells, CD3^+^CD4^+^ T cells, CD3^+^CD8^+^ T cells, B cells, and NK cells, while another flow antibody panel with FITC-CD4, AC7-CD8, PC5.5-CD45RA, and PC7-CD62L was applied to classify CD4^+^ Tn cells, CD4^+^ Tef cells, CD4^+^ Tem cells, CD4^+^ Tcm cells, CD8^+^ Tn cells, CD8^+^ TefCD8^+^ Tem cells, and CD8^+^ Tcm cells. After centrifugation, the washed cells were resuspended in 200 μL of PBS and transferred to a 96-well plate containing a mitochondria-specific dye APC (UBBIO, Zhejiang, China) to assess SCMM and MMP-Low. The samples were incubated in a consistently controlled environment at 37°C for 30 min (protected from light). The MMP-Low proportion was recorded based on the group of cells with low APC fluorescence intensity, and MM was measured using the median fluorescence intensity (MFI) of APC (Puleston, 2015; Yu et al., 1999). SCMM was calculated using a built-in algorithm based on the MFI and the counts of cell subsets. All samples were tested using a Cytek® Aurora cytometer (Cytek Biosciences, 26 Fremont, CA).

### Plasma total DNA extraction and quantification of circulating mtDNA copy numbers

Thawed plasma was centrifuged at 10,000 g for 10 min. Total DNA was extracted from 200 µL plasma using the M5 CWhipro Circulating Nucleic Acid Kit (MF063-plus-05, Mei5bio, Beijing, China), according to the manufacturer’s protocol for plasma/serum. The extracted DNA was then eluted in 30 μL of elution buffer.

The circulating mtDNA level was measured using the 2X M5 HiPer SYBR Premix EsTaq (with Tli RNaseH) qPCR assay (MF787-01, Mei5bio, Beijing, China) with a Roche LightCycler 480 System. MT-ND2 (mitochondrial-encoded NADH: ubiquinone oxidoreductase core subunit 2) was amplified to reflect the levels of circulating mtDNA. The primer sequences were as follows: MT-ND2 (forward: 5′-CACAGAAGCTGCCATCAAGTA-3′; reverse 5′-CCGGAGAGTATATTGTTGAAGAG-3′) (Sangon Biotech, Beijing, China). PCR standards for MT-ND2 (90 bp) were generated by cloning complementary DNA sequences in plasmid PUC57 (GenScript Co. Ltd., Nanjing, China). Concentrations were converted to copy numbers using the formula mol/g x molecules/mol = molecules/g via a DNA copy number calculator online website (http://cels.uri.edu/gsc/cndna.html; University of Rhode Island Genomics and Sequencing Center) (Nga et al., 2010; Zozaya-Hinchliffe et al., 2008). Plasmid DNA (MT-ND2) solutions were diluted in 10-fold serial dilutions and used as standards.

All samples were analyzed in duplicate, and a no-template control was included in each analysis. The analyses of circulating mtDNA levels were expressed in copies per microliter of plasma based on the following calculation (Chiu et al., 2003):

$$c=Q\times V_{\text{DNA}}/V_{\text{PCR}}\times 1/V_{\text{ext}},$$

where c is the concentration of DNA in plasma (copies/μL plasma); Q is the quantity (copies) of DNA determined using the sequence detector in a PCR; V_DNA_ is the total volume of plasma DNA solution obtained after extraction, typically 200 mL per extraction; V_PCR_ is the volume of the plasma DNA solution used for PCR, typically 2 μL of a 10-fold diluted plasma DNA solution; and V_ext_ is the volume of plasma extracted, typically 30 μL.

### Statistical analysis

SPSS 23.0 software and GraphPad Prism 9 were used for statistical analysis. Categorical variables were shown as percentages. The normality of data distribution was evaluated using the Shapiro–Wilk test. Normal distribution data were presented as mean ± SD, whereas non‐normally distributed data were expressed as median (IQR). Student’s t-tests or Mann–Whitney U-tests were performed to analyze the differences in continuous variables between patients and controls. In addition, the χ^2^ test was used to detect the count data. Spearman’s correlation test was used to assess the possible relationship between the level of mitochondrial damage in immune cells, the laboratory examination value, and the SLEDAI-2K score. The receiver operating characteristic (ROC) curves were evaluated for the diagnostic value of mitochondrial damage indicators. Two-tailed *P* < 0.05 was indicated as statistically significant. G*Power 3.1 software was used to conduct a *post hoc* power analysis for each significant parameter to determine whether the available sample sizes provided sufficient power to detect statistically significant effects. Hiplot (https://hiplot.org) and GraphPad Prism 9 were used to visualize the data.

## Results

### Characteristics of study individuals

A total of 52 patients with SLE and 30 HCs were enrolled in our study. The detailed demographic and clinical characteristics of all individuals are shown in Table 1. Patients with SLE included 49 female individuals and 3 male individuals, whose median age was 34 years old (IQR: 27.25–44.00 years old). The HCs included 27 female individuals and 3 male individuals, with an age of 34.6 ± 8.6 years. The median SLEDAI-2K at the time of sample collection was 8. The levels of lymphocyte count, hemoglobin, and platelets were significantly decreased in patients with SLE compared to HCs, while the levels of blood urea nitrogen, blood urea acid, and C-reactive protein were significantly increased (Table 1). Seventeen patients with SLE were grouped into inactive SLE, and 35 patients with SLE were grouped into active SLE according to SLEDAI-2K.

**TABLE 1:** Demographic and clinical characteristics of the study individuals.

Characteristic	SLE (n = 52)	HC (n = 30)	P value
Age, years	34.00 (27.25–44.00)	35.03 ± 9.46	0.821
WBC count, ×10^9^/L	5.29 (4.22–7.49)	5.50 ± 1.41	0.897
Lymphocyte, ×10^12^/L	1.09 (0.79–1.80)	1.90 ± 0.51	<0.001
Hemoglobin, g/L	111.70 ± 23.75	133.67 ± 8.91	<0.001
Platelets, ×10^9^/L	194.02 ± 83.19	260.27 ± 67.97	<0.001
Creatinine, µmol/L	66.00 (54.00–93.75)	63.77 ± 6.68	0.658
BUN, mmol/L	5.56 (3.74–9.29)	4.52 ± 0.82	0.035
UA, µmol/L	359.00 (299.25–459.50)	282.43 ± 48.13	<0.001
CRP, mg/L	1.05 (0.37–3.08)	0.39 (0.26–1.05)	0.003
C3, g/L	0.69 ± 0.29	NA	
C4, g/L	0.10 ± 0.07	NA	
IgG, g/L	12.55 (10.03–15.83)	NA	
IgA, g/L	2.46 ± 1.20	NA	
IgM, g/L	0.94 (0.66–1.25)	NA	
Anti-dsDNA, IU/mL	49.00 (10.60–106.25)	NA	
Urinary proteins, mg/L	0.92 (0.14–4.90)	NA	
ESR, mm/h	24.50 (8.75–45.00)	NA	
SLEDAI-2K	8 (4–17)	NA	

SLE, systemic lupus erythematosus; HCs, healthy controls; WBC, white blood cell; BUN, blood urea nitrogen; UA, blood urea acid; ESR, erythrocyte sedimentation rate; SLEDAI-2K, Systemic Lupus Erythematosus Disease Activity Index 2000.

### Differences in mitochondrial impairment in T cells, B cells, and NK cells between patients with SLE and HCs

**FIGURE 1:**
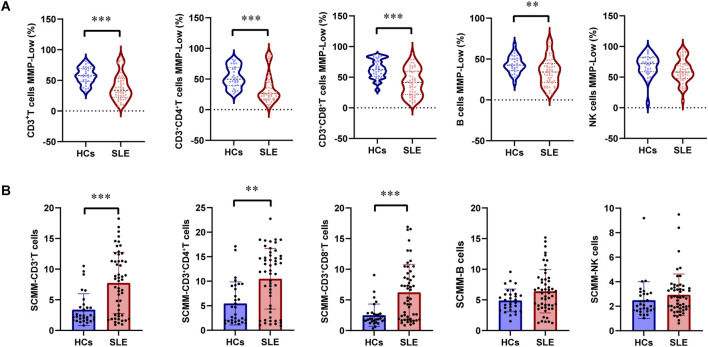
Changes in MMP-Low and SCMM in T, B, and NK lymphocytes between patients with SLE and HCs. **(A)** Proportional changes in the subsets of T, B, and NK lymphocytes with MMP-Low. MMP-Low was calculated based on the group of cells using low APC fluorescence intensity. MMP-Low was normalized across cell types based on the logit correction model: log(Y) = nlog(X)+a. The detailed normalization method has been granted an invention patent by the China National Intellectual Property Administration, with the patent publication number CN114577774B. **(B)** Comparison of SCMMs in T, B, and NK lymphocytes between patients with SLE and HCs. MM was measured using the median fluorescence intensity of APC, and SCMM of lymphocytes was obtained by calculating the absolute count of cells and MM. Statistically significant differences were obtained in the MMP-Low and SCMM in lymphocyte subsets among the SLE group (n = 52) and HC group (n = 30). *P* values were determined using Student’s t-tests or Mann–Whitney U-tests. *P* < 0.05 indicated statistical significance. *P < 0.05, **P < 0.01, and ***P < 0.001.

The proportions of CD3^+^T cells (*P* = 0.005) and CD3^+^CD8^+^T cells (*P* < 0.001) were significantly higher in patients with SLE than in HCs, while the proportion of NK cells (*P* < 0.001) was significantly lower in patients with SLE than in HCs (Table 2). The gating strategy for the fluorescence intensity of mitochondrial staining of T cells, B cells, and NK cells is shown in Supplementary Figure S1. MMP^−^Low proportions were used to reflect the MM of lymphocytes. The percentages of CD3^+^T cell MMP^−^Low (*P* < 0.001, Figure 1A), CD3^+^CD4^+^ T cell MMP-Low (*P* < 0.001, Figure 1A), CD3^+^CD8^+^ T cells (*P* < 0.001, Figure 1A), and B cells (*P* = 0.006, Figure 1A) were significantly lower in patients with SLE than in HCs. In addition, SCMM was calculated based on the MFI and the counts of cell subsets to reflect the MM of each single cell subset in their subsets. We observed that the SCMMs of CD3^+^ T cells (*P* < 0.001, Figure 1B), CD3^+^CD4^+^ T cells (*P* = 0.001, Figure 1B), and CD3^+^CD8^+^ T cells (*P* < 0.001, Figure 1B) were significantly higher in patients with SLE than in HCs, while no significant difference was detected in SCMMs of B cells (*P* = 0.057, Figure 1B) and NK cells (*P* = 0.121, Figure 1B) between patients with SLE and HCs. Meanwhile, the statistical power for SCMM-CD3^+^ T cells, SCMM-CD3^+^CD4^+^ T cells, and CD3^+^CD8^+^ T cells was found to be greater than 0.8 (Supplementary Table S1), indicating that the sample sizes for these results were sufficiently powered.

**TABLE 2:** Comparison of parameters between patients with SLE and HCs.

Parameter	SLE (n = 52)	HCs (n = 30)	P value
CD3^+^T cells (% of lymphocytes)	72.64 (70.10–83.44)	68.52 ± 8.67	0.005
CD3^+^CD4^+^T cells (% of CD3^+^T cells)	32.10 ± 9.16	34.12 (30.93–36.53)	0.214
CD3^+^CD8^+^T cells (% of CD3^+^T cells)	38.93 ± 12.14	27.74 ± 5.18	<0.001
B cells (% of lymphocytes)	12.75 ± 7.50	10.65 ± 3.53	0.345
NK cells (% of lymphocytes)	7.84 (3.81–12.07)	17.21 ± 9.19	<0.001
CD4^+^Tn (% of CD4^+^T cells)	35.75 ± 15.09	42.72 ± 13.71	0.04
CD4^+^Tef (% of CD4^+^T cells)	2.99 (1.30 ± 6.95)	4.23 ± 2.99	0.658
CD4^+^Tem (% of CD4^+^T cells)	18.95 (18.35–31.38)	20.16 ± 9.75	0.386
CD4^+^Tcm (% of CD4^+^T cells)	38.93 ± 12.43	32.88 ± 8.41	0.017
CD8^+^Tn (% of CD8^+^T cells)	35.69 ± 17.63	36.57 ± 15.16	0.819
CD8^+^Tef (% of CD8^+^T cells)	22.87 ± 12.80	18.90 ± 8.28	0.256
CD8^+^Tem (% of CD8^+^T cells)	26.92 ± 13.17	30.25 ± 11.42	0.251
CD8^+^Tcm (% of CD8^+^T cells)	12.59 (9.01–17.89)	14.27 ± 6.13	0.832
MMP-Low CD3^+^T cells (%)	38.90 ± 21.28	57.55 ± 13.45	<0.001
MMP-Low CD3^+^CD4^+^T cells (%)	26.18 (17.26–44.36)	52.98 ± 17.40	<0.001
MMP-Low CD3^+^CD8^+^T cells (%)	44.71 ± 22.05	63.24 ± 15.03	<0.001
MMP-Low B cells (%)	34.51 ± 17.00	43.21 ± 10.35	0.006
MMP-Low NK cells (%)	60.30 ± 19.44	68.51 ± 17.78	0.061
MMP-Low CD4^+^Tn (%)	26.48 (8.35–53.35)	68.86 (42.26–88.71)	<0.001
MMP-Low CD4^+^Tef (%)	44.61 ± 29.71	29.54 (8.27–56.46)	0.125
MMP-Low CD4^+^Tem (%)	32.29 (23.16–51.78)	52.60 ± 15.31	0.002
MMP-Low CD4^+^Tcm (%)	15.66 (9.58–38.37)	45.23 (23.62–74.86)	<0.001
MMP-Low CD8^+^Tn (%)	47.01 ± 23.18	74.33 ± 13.65	<0.001
MMP-Low CD8^+^Tef (%)	33.50 ± 22.76	50.12 ± 19.17	<0.001
MMP-Low CD8^+^Tem (%)	22.99 (15.15–40.37)	55.78 ± 15.68	<0.001
MMP-Low CD8^+^Tcm (%)	13.62 (7.90–27.98)	39.06 ± 14.06	<0.001
SCMM-CD3^+^T cells	8.18 (2.47–11.33)	2.32 (1.70–3.90)	<0.001
SCMM-CD3^+^CD4^+^T cells	11.94 (3.87–15.06)	3.97 (1.95–9.10)	0.001
SCMM-CD3^+^CD8^+^T cells	5.27 (1.90–9.21)	1.89 (1.56–2.77)	<0.001
SCMM-B cells	6.41 ± 3.54	4.92 ± 1.86	0.057
SCMM-NK cells	2.71 (1.84–3.52)	2.06 (1.67–3.02)	0.121
SCMM-CD4^+^Tn	9.52 (2.97–15.84)	2.32 (1.64–6.68)	<0.001
SCMM-CD4^+^Tef	15.79 (2.03–26.38)	22.24 (2.92–26.49)	0.544
SCMM-CD4^+^Tem	9.71 (4.46–14.18)	3.14 (2.49–9.44)	0.006
SCMM-CD4^+^Tcm	12.46 ± 6.80	5.76 (2.19–11.08)	<0.001
SCMM-CD8^+^Tn	4.39 (2.62–8.92)	1.88 (1.48–2.45)	<0.001
SCMM-CD8^+^Tef	9.51 ± 7.08	6.27 ± 4.19	0.066
SCMM-CD8^+^Tem	9.73 ± 5.39	3.00 (1.91–6.51)	<0.001
SCMM-CD8^+^Tcm	13.10 ± 6.45	8.92 ± 5.08	0.003

NK cells, natural killer cells; Tn, T naïve; Tef, T effector; Tem, T effector memory; Tcm, T central memory; MMP-Low, low mitochondrial membrane potential; SCMM, single-cell mitochondrial mass.

### Differences in mitochondrial impairment in T-cell subsets between patients with SLE and HCs

**FIGURE 2:**
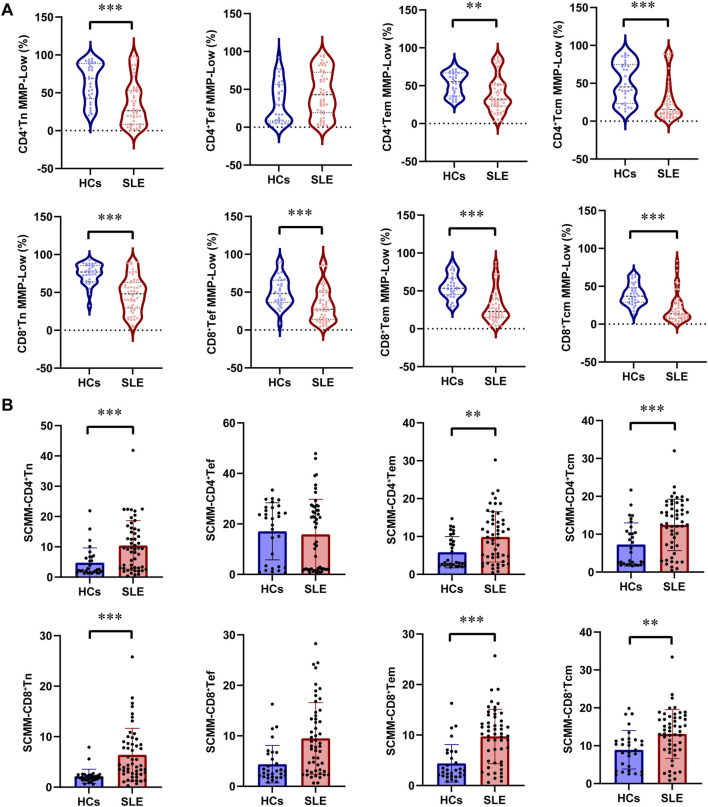
Changes in MMP-Low and SCMM in CD4 and CD8 lymphocyte subsets between patients with SLE and HCs. **(A)** Proportional changes in the subsets of CD4 and CD8 lymphocytes with MMP-Low. MMP-Low was calculated based on the group of cells using low APC fluorescence intensity. MMP-Low was normalized across cell types based on the logit correction model: log(Y) = nlog(X)+a. The detailed normalization method has been granted an invention patent by the China National Intellectual Property Administration, with the patent publication number CN114577774B. **(B)** Comparison of SCMM in CD4 and CD8 lymphocyte subsets between patients with SLE and HCs. MM was measured using the median fluorescence intensity of APC, and SCMM of lymphocytes was obtained by calculating the absolute count of cells and MM. Statistically significant differences were obtained in the MMP-Low and SCMM in lymphocyte subsets among the SLE group (n = 52) and HC group (n = 30). *P* values were determined using Student’s t-tests or Mann–Whitney U-tests. *P* < 0.05 indicated statistical significance. *P < 0.05, **P < 0.01, and ***P < 0.001.

Further analysis of mitochondrial impairment in T-cell subsets was detected in patients with SLE and HCs. First, the percentage of CD4^+^ Tn was significantly decreased in patients with SLE compared with HCs (*P* = 0.04, Table 2), while the percentage of CD4^+^ Tcm was significantly increased in patients with SLE and HCs (*P* = 0.017, Table 2). However, the power for the percentage of CD4^+^ Tn and CD4^+^ Tcm cells was found to be less than 0.8 (Supplementary Table S1), indicating an insufficiently powered sample size for these two parameters. No significant alternation of subsets in CD8^+^ T cells was observed between patients with SLE and HCs (Table 2). The gating strategy for the fluorescence intensity of mitochondrial staining of T-cell subsets is shown in Supplementary Figure S2. In CD4^+^ T cell subsets, the percentages of CD4^+^Tn MMP-Low (*P* < 0.001), CD4^+^Tem MMP-Low (*P* = 0.002), and CD4^+^ Tcm MMP-Low (*P* < 0.001) were significantly lower in patients with SLE than in HCs (Figure 2A). However, the SCMMs of CD4^+^Tn, CD4^+^Tem, and CD4^+^Tcm cells were significantly higher in patients with SLE than in HCs (Figure 2B). In CD8^+^T cell subsets, the percentages of CD8^+^Tn MMP-Low (*P* < 0.001), CD8^+^ Tef MMP-Low (*P* < 0.001), CD8^+^Tem MMP-Low (*P* < 0.001), and CD8^+^Tcm MMP-Low (*P* < 0.001) were significantly decreased in patients with SLE (Figure 2A). In addition, the SCMMs of CD8^+^Tn (*P* < 0.001), CD8^+^Tem (*P* < 0.001), and CD8^+^Tcm (*P* = 0.003) cells indicated a significant increase in patients with SLE compared with HCs (Figure 2B). The *post hoc* power analysis showed that the power for both the significant MMP-Low and SCMM of CD4^+^ and CD8^+^ T-cell subsets was sufficient to detect statistically significant effects given the available sample sizes (Supplementary Table S1).

### Comparison of circulating mtDNA in patients with SLE and HCs

**FIGURE 3:**
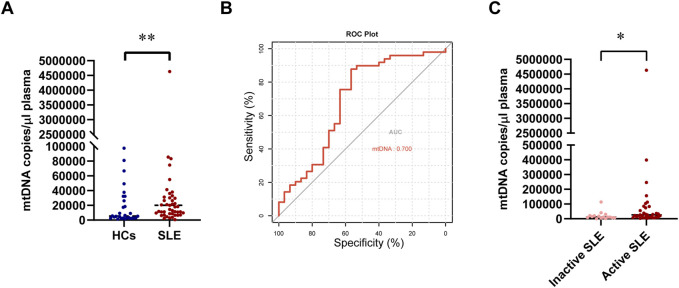
Changes in circulating mtDNA levels between patients with SLE and HCs. **(A)** Comparison of circulating mtDNA levels between patients with SLE and HCs. **(B)** Receiver operating characteristic curve for mtDNA plasma concentrations for distinguishing patients with SLE from HCs. **(C)** Comparison of circulating mtDNA levels between inactive and active SLE. Statistically significant differences were obtained in mtDNA levels between the SLE group (n = 49) and HC group (n = 30), as well as between the inactive SLE group (n = 16) and the active SLE group (n = 33). *P* values were determined using the Mann–Whitney U-tests. *P* < 0.05 indicated statistical significance. *P < 0.05, **P < 0.01, and ***P < 0.001.

The circulating mtDNA (ND2) was detected in 49 patients with SLE and 30 healthy controls. The circulating mtDNA levels were significantly higher in patients with SLE than in HCs (median 20,025 copies/ul *versus* 5,448.75 copies/μL, *P* = 0.004; Figure 3A). We further analyzed all 79 subjects to evaluate the diagnostic value of circulating mtDNA levels in distinguishing patients with SLE from HCs. The ROC curve analysis of circulating mtDNA revealed an area under the curve (AUC) of 0.700 (P = 0.0048, Figure 3B). This analysis exhibited a sensitivity of 87.8% and a specificity of 56.7%. Furthermore, we observed that the circulating mtDNA was significantly higher in active SLE than in inactive SLE (median 26,325 copies/μL *versus* 11,175 copies/μL, *P* = 0.036; Figure 3C).

### Diagnostic value of mitochondrial impairment in lymphocytes for SLE

**FIGURE 4:**
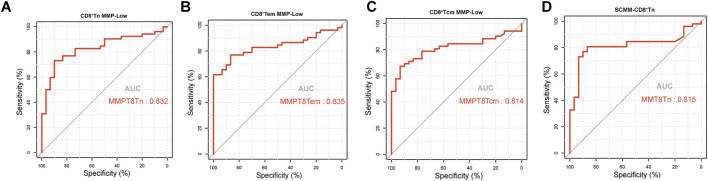
Receiver operating characteristic curve for CD8^+^Tn MMP-Low **(A)**, CD8^+^Tem MMP-Low **(B)**, CD8^+^Tcm MMP-Low **(C)**, and SCMM-CD8^+^Tn **(D)** for distinguishing patients with SLE (n = 52) from HCs (n = 30).

The ROC curve analysis was performed to evaluate the utility of mitochondrial impairment quantification in distinguishing patients with SLE from HCs. CD8^+^ Tn MMP-Low (AUC = 0.832, *P* < 0.001; Figure 4A), CD8^+^Tem MMP-Low (AUC = 0.835, *P* < 0.001, Figure 4B), CD8^+^ Tcm MMP-Low (AUC = 0.81, *P* < 0.001; Figure 4C), and SCMM-CD8^+^ Tn (AUC = 0.815, *P* < 0.001; Figure 4D) cells showed a moderate diagnostic value for SLE, as indicated by their AUC values exceeding 0.8 (Table 3). However, CD4^+^ Tef MMP-Low (AUC = 0.602, *P* = 0.124), SCMM-B cells (AUC = 0.626, *P* = 0.0579), SCMM-NK cells (AUC = 0.603, *P* = 0.121), and SCMM-CD4^+^ Tef (AUC = 0.54, *P* = 0.5422) cells showed no diagnostic value for SLE (Table 3).

**TABLE 3:** AUCs for the assessed variables in patients with SLE and HCs.

Parameter	Sensitivity (%)	Specificity (%)	AUC	P value
CD3^+^T cells MMP-Low	67.3	83.3	0.781	<0.001
CD3^+^CD4^+^T cells MMP-Low	61.5	96.7	0.772	<0.001
CD3^+^CD8^+^T cells MMP-Low	63.5	90	0.775	<0.001
B cells MMP-Low	55.8	80	0.683	0.0059
NK cells MMP-Low	53.8	76.7	0.649	0.0249
CD4^+^Tn MMP-Low	82.7	60	0.766	<0.001
CD4^+^Tef MMP-Low	78.8	46.7	0.602	0.124
CD4^+^Tem MMP-Low	53.8	86.7	0.705	0.0021
CD4^+^Tcm MMP-Low	51.9	96.7	0.759	<0.001
CD8^+^Tn MMP-Low	73.1	90	0.832	<0.001
CD8^+^Tef MMP-Low	51.9	93.3	0.733	<0.001
CD8^+^Tem MMP-Low	76.9	86.7	0.835	<0.001
CD8^+^Tcm MMP-Low	67.3	93.3	0.814	<0.001
SCMM-CD3^+^T cells	67.3	80	0.744	<0.001
SCMM-CD3^+^CD4^+^T cells	55.8	93.3	0.717	0.0011
SCMM-CD3^+^CD8^+^T cells	63.5	83.3	0.747	<0.001
SCMM-B cells	46.1	86.7	0.626	0.0579
SCMM-NK cells	40.3	86.7	0.603	0.121
SCMM-CD4^+^Tn	61.5	83.3	0.74	<0.001
SCMM-CD4^+^Tef	38.4	80	0.54	0.5442
SCMM-CD4^+^Tem	76.9	56.7	0.685	0.006
SCMM-CD4^+^Tcm	65.4	76.7	0.733	<0.001
SCMM-CD8^+^Tn	80.8	86.7	0.815	<0.001
SCMM-CD8^+^Tef	36.5	90	0.622	<0.001
SCMM-CD8^+^Tem	71.2	86.7	0.789	<0.001
SCMM-CD8^+^Tcm	63.5	83.3	0.699	0.003

AUC, area under the curve; NK cells, natural killer cells; Tn, T naïve; Tef, T effector; Tem, T effector memory; Tcm, T central memory; MMP-Low, low mitochondrial membrane potential; SCMM, single-cell mitochondrial mass.

### Correlation analysis of mitochondrial impairment in lymphocytes and disease activity in patients with SLE

**FIGURE 5:**
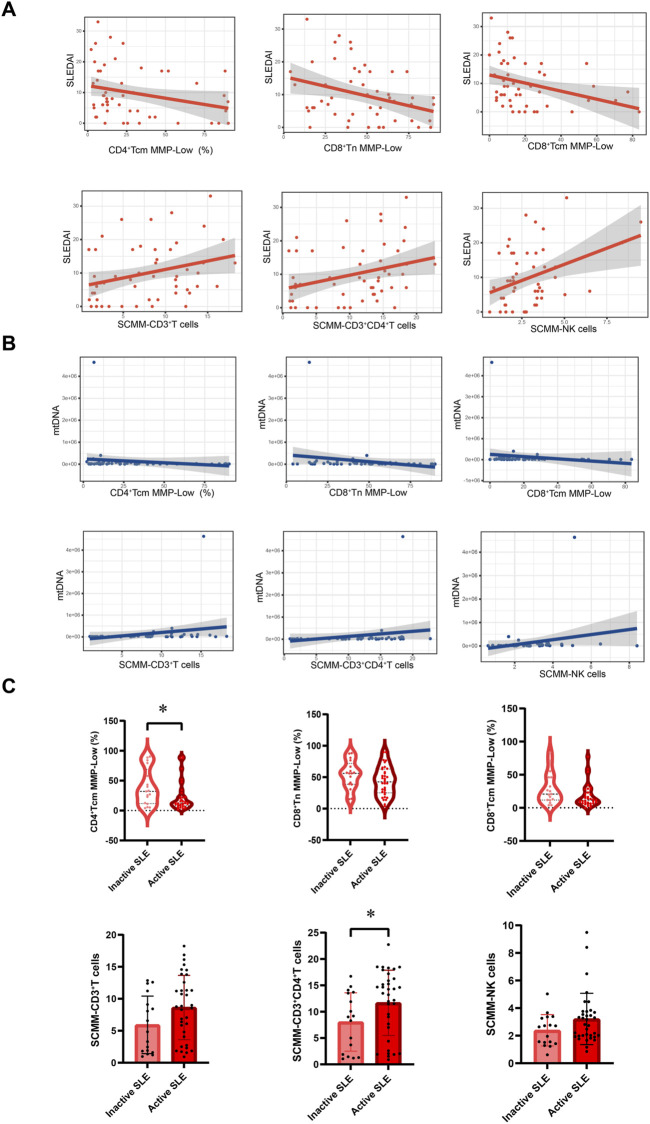
Association between mitochondrial impairment parameters and disease activity of SLE. **(A)** Association between CD4^+^Tcm cell MMP-Low, CD8^+^Tn cell MMP-Low, CD8^+^Tcm cell MMP-Low, SCMM-CD3^+^T cells, SCMM-CD3^+^CD4^+^T cells, SCMM-NK cells, and SLEDAI-2K scores. **(B)** Association between CD4^+^Tcm cell MMP-Low, CD8^+^Tn cell MMP-Low, CD8^+^Tcm cell MMP-Low, SCMM-CD3^+^T cells, SCMM-CD3^+^CD4^+^T cells, SCMM-NK cells, and circulating mtDNA levels. **(C)** Comparison of CD4^+^Tcm cell MMP-Low, CD8^+^Tn cell MMP-Low, CD8^+^Tcm cell MMP-Low, SCMM-CD3+T cells, SCMM-CD3^+^CD4+T cells, and SCMM-NK cells between inactive and active SLE groups. Statistically significant differences were obtained in CD4+Tcm cell MMP-Low, CD8+Tn cell MMP-Low, CD8+Tcm cell MMP-Low, SCMM-CD3^+^T cells, SCMM-CD3^+^CD4^+^T cells, and SCMM-NK cells between the inactive SLE group (n = 17) and active SLE group (n = 35). *P* values were determined using Student’s t-tests, Mann–Whitney U-tests, and Spearman’s correlation test. *P* < 0.05 indicated statistical significance. *P < 0.05, **P < 0.01, and ***P < 0.001.

Spearman’s correlation test showed that NK cell MMP-Low was positively associated with C3 (r = 0.401, *P* = 0.003; Supplementary Figure S3) and C4 (r = 0.301, *P* = 0.03; Supplementary Figure S3) at the same time. In addition, CD3^+^ T cell MMP-Low (r = 0.333, *P* = 0.016; Supplementary Figure S3) and CD3^+^CD8^+^ T cell MMP-Low (r = 0.328, *P* = 0.018; Supplementary Figure S3) were positively associated with C3 in patients with SLE, while SCMM-CD3^+^ T cells (r = 0.-309, *P* = 0.026; Supplementary Figure S3), SCMM-CD3^+^CD4^+^ T cells (r = −0.339, *P* = 0.014; Supplementary Figure S3), and SCMM-CD3^+^CD8^+^ T cells (r = −0.324, *P* = 0.019; Supplementary Figure S3) was negatively associated with C3. CD4^+^ Tcm MMP-Low (SLEDAI-2K: r = −0.303, *P* = 0.029, Figure 5A; mtDNA: r = −0.387, *P* = 0.006, Figure 5B), CD8^+^ Tn MMP-Low (SLEDAI-2K: r = −0.359, *P* = 0.009, Figure 5A; mtDNA: r = −0.320, *P* = 0.025, Figure 5B), and CD8^+^ Tcm MMP-Low (SLEDAI-2K: r = −0.298, *P* = 0.032, Figure 5A; mtDNA: r = −0.290, *P* = 0.043; Figure 5B) were negatively associated with the SLEDAI-2K and circulating mtDNA in patients with SLE, while SCMM-CD3^+^ T cells (SLEDAI-2K: r = 0.295, *P* = 0.034, Figure 5A; mtDNA: r = 0.436, *P* = 0.002, Figure 5B), SCMM-CD3^+^CD4^+^ T cells (SLEDAI-2K: r = 0.355, *P* = 0.01 Figure 5A; mtDNA: r = 0.462, *P* = 0.001, Figure 5B), and SCMM-NK cells (SLEDAI-2K: r = 0.300, *P* = 0.031, Figure 5A; mtDNA: r = 0.326, *P* = 0.022, Figure 5B) was positively associated with the SLEDAI-2K and circulating mtDNA. Furthermore, we observed that CD4^+^ Tcm MMP-Low was significantly decreased in active SLE (*P* = 0.0397, Figure 5C), and SCMM-CD3^+^CD4^+^ T cells was significantly increased in active SLE compared with inactive SLE (*P* = 0.025, Figure 5C).

## Discussion

This cross-sectional study revealed a higher alteration in MM in some subsets of lymphocyte subsets in patients with SLE, indicating increased mitochondrial biogenesis or heightened cellular activation in SLE. In addition, the level of circulating mtDNA was elevated in patients with SLE compared with HCs. Meanwhile, we observed that MM in some subsets of lymphocytes was positively associated with the level of circulating mtDNA and SLEDAI-2K in patients with SLE. Our data established that abnormal MM in peripheral lymphocyte subsets might be involved in the pathogenesis of SLE.

MM is usually defined as the sum of the masses of all mitochondria and their contents within a cell (Yang et al., 2019). MM homeostasis contributes to maintaining normal mitochondrial function, which depends on the mitochondrial quality control encompassing processes like mitophagy, mitochondrial biogenesis, and mitochondrial dynamics (Miwa et al., 2022). Increased MM suggests mitophagy dysfunction (Chourasia et al., 2015), enhanced mitochondrial biogenesis (Valero, 2014), and imbalanced mitochondrial fusion–fission.

Mitophagy could contribute to eliminating dysfunctional mitochondria and helps the cell respond to hypoxia and nutrient starvation by decreasing MM (Chourasia et al., 2015). Some studies showed that insufficient mitophagy could appear in T cells of SLE patients, resulting in increased MM and contributing to immune dysregulation in SLE (Caza et al., 2014; Xu et al., 2020). Dysregulated type I interferon (IFN) signaling is a hallmark of SLE (Caielli et al., 2016), contributing to the breakdown of immune tolerance and the maintenance of autoimmune reactions in SLE (Eloranta and Rönnblom, 2016). The loss of mitophagy can lead to the accumulation of ROS and mtDNA, which, in turn, promotes the release of type I IFNs (Halfon et al., 2024; Harris et al., 2018). Activated IFNα signaling in SLE monocytes leads to increased mitochondrial oxidative stress, characterized by elevated levels of ROS and mtDNA (Gkirtzimanaki et al., 2018). Additionally, another study has shown that SLE CD8^+^ T cells with a high type I IFN signature exhibit enlarged mitochondria and impaired mitochondrial metabolism, including reduced spare respiratory capacity (Buang et al., 2021). Therefore, mitochondrial dysfunction resulting from impaired mitophagy may be linked to the IFN signature observed in patients with SLE.

Mitochondrial biogenesis is the process of producing new mitochondrial offspring to maintain an adequate mitochondrial number and could increase MM and metabolic capacity in cells (Chang et al., 2022; Ding et al., 2021). Increased mitochondrial biogenesis can also be observed in T cells of SLE patients, contributing to the excessive production of reactive oxygen intermediates (ROIs) and the spread of oxidative stress (Perl, 2013). Oxidative stress can cause abnormal activation of cell-death signals and further trigger the release of nuclear debris from apoptotic and necrotic cells, thereby promoting the production of autoantibodies and causing immune system dysfunction, which participates in the pathogenesis of SLE (Perl, 2013; Perl et al., 2011).

Mitochondria constantly maintain a dynamic balance between local fission and fusion events to ensure structural stability and sufficient energy production for cellular metabolism (Ding et al., 2021). However, unbalanced fission and fusion events can alter MM and cause mitochondrial dysfunction (Yu et al., 2020). Dynamin-related protein 1 (Drp1) is a key molecule involved in mitochondrial fission and mitophagy (Halfon et al., 2024; Buang et al., 2021). The depletion of Drp1 can lead to increased mitochondrial fusion, impaired mitophagy, and increased MM (Caza et al., 2014). In peripheral blood lymphocytes of patients with SLE, Drp1 levels are significantly decreased compared to those in HCs. This reduction in Drp1 leads to mitochondrial hyperactivation, characterized by enlarged mitochondria, increased MM, and disrupted mitochondrial homeostasis (Caza et al., 2014).

Therefore, increased MM might reflect mitochondrial dysfunction and the alteration of cell function. In this study, SCMM was calculated by detecting the MFI of lymphocyte subsets using MitoTracker’s fluorescent probe by flow cytometry (Pang et al., 2022). It was then divided by the count of the corresponding lymphocyte subsets to obtain the SCMM of each lymphocyte subset (Pang et al., 2022). MMP refers to the voltage difference between the inner and outer mitochondrial membranes, reflecting the ability to synthesize ATP (Lee et al., 2002). Increased MMP levels indicate mitochondrial hyperpolarization (MHP). Persistent MHP can occur in T cells of SLE patients, leading to increased ROI production, mitochondrial biogenesis, and MM (Gergely et al., 2002; Nagy et al., 2004). Meanwhile, elevated MMP levels may inhibit mitophagy (Wan et al., 2022). Therefore, these two markers may reflect mitochondrial disturbance and mitochondrial energy metabolism in immune cells (Hu et al., 2022; Zhou et al., 2024).

It has been reported that the imbalanced distribution of circulating lymphocyte subsets has existed in patients with SLE (Lee et al., 2002; Torres-Ruiz et al., 2018; Picca et al., 2017). In our study, we observed that the proportion of CD3^+^CD8^+^T cells was increased in patients with SLE compared to HCs, while the proportion of NK cells was decreased. CD3^+^CD8^+^T cells and NK cells are important cytotoxic cells. However, Li et al. (2022) reported that the reduced cytolytic activity in CD3^+^CD8^+^T cells among patients with SLE results in higher rates of infection and the sustenance of autoimmunity. Patients with SLE exhibited elevated SCMM and decreased MMP-low in CD3^+^CD8^+^T cells in this study, indicating that mitochondrial dysfunction was observed in CD3^+^CD8^+^T cells of patients with SLE. Buang et al. (2021) also observed that CD8^+^ T cells with enlarged mitochondria and lower spare respiratory capacity in patients with SLE, which is associated with type I IFN exposure in SLE. Meanwhile, the cytotoxicity of NK cells is markedly suppressed in patients with SLE, contributing to immune system dysregulation in SLE (Park et al., 2009; Luo et al., 2022). However, no mitochondrial abnormalities were found in NK cells of patients with SLE because no significant differences in SCMM and MMP-Low of NK cells were observed in patients with SLE and HCs. Thus, the potential reason for NK cell impairment might not be related to mitochondrial dysfunction in patients with SLE. Although the distribution of CD3^+^CD4^+^T cells was not altered in patients with SLE, both SCMM and MMP-Low in CD3^+^CD4^+^T cells showed a significant difference between patients with SLE and HCs. Previous studies also reported that increased MM and hyperpolarization in CD4^+^T cells of patients with SLE lead to mitochondrial dysfunction (Caza et al., 2012).

CD3^+^CD4^+^T cells and CD3^+^CD8^+^T cells were further subdivided into subsets based on the expression of CD45RA and CD62L. The proportion of CD4^+^Tn cells was significantly decreased in patients with SLE compared to HCs, while the proportion of CD4^+^ Tcm cells was significantly increased in this study. This result is consistent with previous studies (Yuan et al., 2022; Sobel et al., 2011; Ugarte-Gil et al., 2015), indicating that the immune system was overactivated in SLE, leading to the process of proliferation and differentiation in naïve T cells. Furthermore, we also observed mitochondrial abnormalities in CD4^+^Tn cells, with higher SCMM and lower MMP-Low levels in patients with SLE compared with HCs, indicating that increased mitochondrial biogenesis and mitochondrial hyperpolarization may promote the activation of immune cells (Perl et al., 2012). The proportions of CD8^+^Tn, CD8^+^Tef, CD8^+^Tem, and CD8^+^Tcm cells showed no significant alteration between patients with SLE and HCs, in accordance with the data from Kajihara et al. (2023). However, CD8^+^ T cell subsets showed mitochondrial abnormalities in patients with SLE. Type I IFN is overproduced in patients with SLE, contributing to breaking the peripheral tolerance. Furthermore, type I IFN affects the mitochondrial function in immune cells by promoting the production of oxidative phosphorylation, especially in memory CD8^+^ T cells (van der Windt et al., 2012). Therefore, the overexpression of type I IFN affects the mitochondrial function of CD8^+^ T cell subsets in patients with SLE.

We observed a significantly higher level of circulating mtDNA in patients with SLE than in HCs, especially in active SLE. This result is consistent with the data from Giaglis et al. (2021). Human mtDNA is a double-stranded and closed-circular DNA molecule located in the mitochondrial matrix, which encodes electron transport chain proteins that are essential for ATP production (Clayton, 1984). The altered mtDNA level contributes to the process of mitochondrial dysfunction by enhancing the immune response (Malik and Czajka, 2013). Meanwhile, we found that MMP-Low and SCMM of some immune cells were associated with the level of circulating mtDNA. In addition, mtDNA also serves as self-antigens that can be recognized by DNA autoantibodies in patients with SLE, which contributes to SLE pathogenesis (Chen and Tsokos, 2022). The release of mtDNA results from defective mitophagy and increased mitochondrial ROS in SLE. Extracellular mtDNA can be recognized as a danger-associated molecular pattern (DAMP) due to its unmethylated CpG sequences, which structurally resemble bacterial DNA; this induces a type I IFN response via the activation of the cyclic GMP-AMP synthase (cGAS)–stimulator of interferon genes (STING) pathway or the endosomal TLR9 pathway (Halfon et al., 2024; Chen and Tsokos, 2022; Lood et al., 2016; West et al., 2015). Type I IFN could further drive B cells to produce autoantibodies in SLE (Hamilton et al., 2018). Therefore, elevated levels of circulating mtDNA play a proinflammatory role in the pathogenesis of SLE. In this study, ROC analysis showed that circulating mtDNA could identify patients with SLE from HCs with high sensitivity, while the specificity and AUC were relatively poorer. Further studies with larger sample sizes are needed to confirm the diagnostic value of circulating mtDNA in SLE.

Previous studies have shown that some immune cell subsets or the co-stimulatory molecule expression on these subsets could be used to diagnose and evaluate disease activity in patients with SLE (Park et al., 2020; Men et al., 2019; Miao et al., 2016; Mortezagholi et al., 2016; Ugarte-Gil et al., 2016). In this study, several parameters demonstrated a good diagnostic value in patients with SLE, including CD8^+^ Tn MMP-Low, CD8^+^ Tem MMP-Low, CD8^+^ Tcm MMP-Low, and SCMM-CD8^+^ Tn. Thus, these indicators may serve as potential biomarkers for SLE diagnosis. Both MMP-Low and SCMM of CD4^+^ T cell subsets did not show good diagnostic performance due to AUC values below 0.8. However, we found that CD4^+^ Tcm MMP-Low and SCMM-CD3^+^CD4^+^ T cells showed a significant but weak correlation with the SLEDAI-2K and circulating mtDNA. This weak correlation suggests that CD4^+^ Tcm MMP-Low and SCMM-CD3^+^CD4^+^ T cells play a limited role in reflecting disease activity. Additionally, both markers showed significant alterations between inactive and active SLE. Therefore, future studies should enroll a larger sample size to validate whether CD4^+^ Tcm MMP-Low and SCMM-CD3^+^CD4^+^ T cells can serve as effective biomarkers for reflecting SLE disease activity.

There are several limitations to this study. First, mitochondrial parameters can be influenced by various biological and environmental factors, such as medication, stress, circadian rhythms, and infections. Our study excluded individuals with infections, but factors like treatment, stress, and circadian rhythms could still act as confounders that might affect mitochondrial parameter results. Future studies should account for these potential confounders to ensure more reliable findings. Second, the sample size was relatively small, and the number of individuals in the HC and SLE groups was not equal, which may reduce the statistical power to detect significant relationships. The *post hoc* power analysis showed that the statistical power for the percentage of CD4^+^Tn, CD4^+^Tcm, and MMP-Low B cells was lower than 0.8, likely due to the limited sample size. This low power increases the risk of Type II errors and may prevent the detection of true difference in these parameters between HC and SLE groups. The small sample size may also be associated with higher variability in the estimates, making the sample statistics less likely to accurately reflect the population parameters. Additionally, the small sample size may introduce selection bias, where the characteristics of the included subjects do not accurately reflect those of the broader population. Therefore, more patients with SLE and HCs should be included in further investigation. Third, this study lacks a validation cohort, and we did not include disease controls to evaluate the utility of mitochondrial alterations in the diagnosis of patients with SLE. This omission indicates that whether these mitochondrial alterations are unique to SLE remains unclear. Meanwhile, we did not compare the diagnostic value of MMP-Low and SCMM in immune cells with existing SLE biomarkers in this study. More studies involving both patients with SLE and patients with other autoimmune diseases are needed to further validate the diagnostic value of MMP-Low and SCMM in immune cells and explore their performance compared to traditional biomarkers. Finally, our study applied a cross-sectional design, which could not reflect the predictive values of MMP-Low and SCMM in immune cells for SLE disease activity and their mechanistic causal roles in the pathogenesis of SLE. Future studies need to use mitochondrial inhibitors or Seahorse metabolic assays to investigate the alteration of the metabolic state in immune cells of SLE.

## Conclusion

In conclusion, our findings indicate that patients with SLE exhibit mitochondrial dysfunction, as evidenced by detecting MMP-Low and SCMM in immune cells and circulating mtDNA levels. The level of mitochondrial impairment in T lymphocytes was significantly more severe in patients with SLE than in HCs. CD8^+^ Tn MMP-Low, CD8^+^ Tem MMP-Low, CD8^+^ Tcm MMP-Low, and SCMM-CD8^+^ Tn cells may serve as potential biomarkers for SLE diagnosis. Furthermore, CD4^+^ Tcm MMP-Low and SCMM-CD3^+^CD4^+^ T cells may reflect the disease activity of patients with SLE. These outcomes help provide novel mitochondrial-based insights into the pathogenesis of SLE.

## Data Availability

The raw data supporting the conclusions of this article will be made available by the authors, without undue reservation.

## Abbreviations

ACR, American College of Rheumatology; ATP, adenosine triphosphate; EDTA, ethylenediaminetetraacetic acid; MM, mitochondrial mass; MMP-Low, low mitochondrial membrane potential; MtDNA, mitochondrial DNA; ROC, receiver operating characteristic; SCMM, single-cell mitochondrial mass; SLE, systemic lupus erythematosus; SLEDAI-2K, Systemic Lupus Erythematosus Disease Activity Index 2000; Tcm, T central memory; Tef, T effector; Tem, T effector memory; Tn, T naïve.
