# Supplementary material for: Mitochondrial mass and mitochondrial membrane potential of peripheral lymphocytes: promising biomarkers of systemic lupus erythematosus
Source: Front Mol Biosci. 2025 Jun 6;12:1585847. doi: 10.3389/fmolb.2025.1585847 (PMC12178850; doi:10.3389/fmolb.2025.1585847)
Supplement: Supplementary file 1 [file Table1.docx]

| **Parameters** | **Statistical tests** | **Tails** | **α** | **effect size** | **Power (1-β)** |
| --- | --- | --- | --- | --- | --- |
| CD3^+^T cells (% of lymphocytes) | Wiclcoxon-Mann-Whitney test | Two | 0.05 | 0.7842052 | 0.9099057 |
| CD3^+^CD8^+^T cells (% of CD3^+^T cells) | t test | Two | 0.05 | 1.198964 | 0.9993268 |
| NK cells (% of lymphocytes) | Wiclcoxon-Mann-Whitney test | Two | 0.05 | 1.050142 | 0.9930345 |
| CD4^+^Tn (% of CD4^+^T cells) | t test | Two | 0.05 | 0.4834731 | 0.5491486 |
| CD4^+^Tcm (% of CD4^+^T cells) | t test | Two | 0.05 | 0.5701044 | 0.6903099 |
| MMP-Low CD3^+^T cells (%) | t test | Two | 0.05 | 1.0477025 | 0.99468 |
| MMP-Low CD3^+^CD4^+^T cells (%) | Wiclcoxon-Mann-Whitney test | Two | 0.05 | 0.8844832 | 0.9609725 |
| MMP-Low CD3^+^CD8^+^T cells (%) | t test | Two | 0.05 | 0.9819898 | 0.9884334 |
| MMP-Low B cells (%) | t test | Two | 0.05 | 0.6181865 | 0.7592486 |
| MMP-Low CD4^+^Tn (%) | Wiclcoxon-Mann-Whitney test | Two | 0.05 | 1.03511 | 0.9917078 |
| MMP-Low CD4^+^Tem (%) | Wiclcoxon-Mann-Whitney test | Two | 0.05 | 0.6664343 | 0.8008449 |
| MMP-Low CD4^+^Tcm (%) | Wiclcoxon-Mann-Whitney test | Two | 0.05 | 0.8639999 | 0.9531215 |
| MMP-Low CD8^+^Tn (%) | t test | Two | 0.05 | 1.43627 | 0.9999882 |
| MMP-Low CD8^+^Tef (%) | t test | Two | 0.05 | 0.78986 | 0.925563 |
| MMP-Low CD8^+^Tem (%) | Wiclcoxon-Mann-Whitney test | Two | 0.05 | 1.352344 | 0.9999044 |
| MMP-Low CD8^+^Tcm (%) | Wiclcoxon-Mann-Whitney test | Two | 0.05 | 1.027776 | 0.9909835 |
| SCMM-CD3^+^T cells | Wiclcoxon-Mann-Whitney test | Two | 0.05 | 1.096723 | 0.9960346 |
| SCMM-CD3^+^CD4^+^T cells | Wiclcoxon-Mann-Whitney test | Two | 0.05 | 0.9376727 | 0.9764759 |
| SCMM-CD3^+^CD8^+^T cells | Wiclcoxon-Mann-Whitney test | Two | 0.05 | 1.071508 | 0.9945972 |
| SCMM-CD4^+^Tn | Wiclcoxon-Mann-Whitney test | Two | 0.05 | 0.8374738 | 0.9945972 |
| SCMM-CD4^+^Tem | Wiclcoxon-Mann-Whitney test | Two | 0.05 | 0.7293672 | 0.8663801 |
| SCMM-CD4^+^Tcm | Wiclcoxon-Mann-Whitney test | Two | 0.05 | 0.8266045 | 0.9355643 |
| SCMM-CD8^+^Tn | Wiclcoxon-Mann-Whitney test | Two | 0.05 | 1.112838 | 0.9967634 |
| SCMM-CD8^+^Tem | Wiclcoxon-Mann-Whitney test | Two | 0.05 | 1.23734 | 0.9994159 |
| SCMM-CD8^+^Tcm | t test | Two | 0.05 | 0.7200003 | 0.925563 |

Supplementary Table 1. The results of post-hoc power analysis for each significant parameter.
