# Supplementary material for: Mitochondrial mass and mitochondrial membrane potential of peripheral lymphocytes: promising biomarkers of systemic lupus erythematosus
Source: Front Mol Biosci. 2025 Jun 6;12:1585847. doi: 10.3389/fmolb.2025.1585847 (PMC12178850; doi:10.3389/fmolb.2025.1585847)
Supplement: Supplementary file 2 [file Image2.pdf]

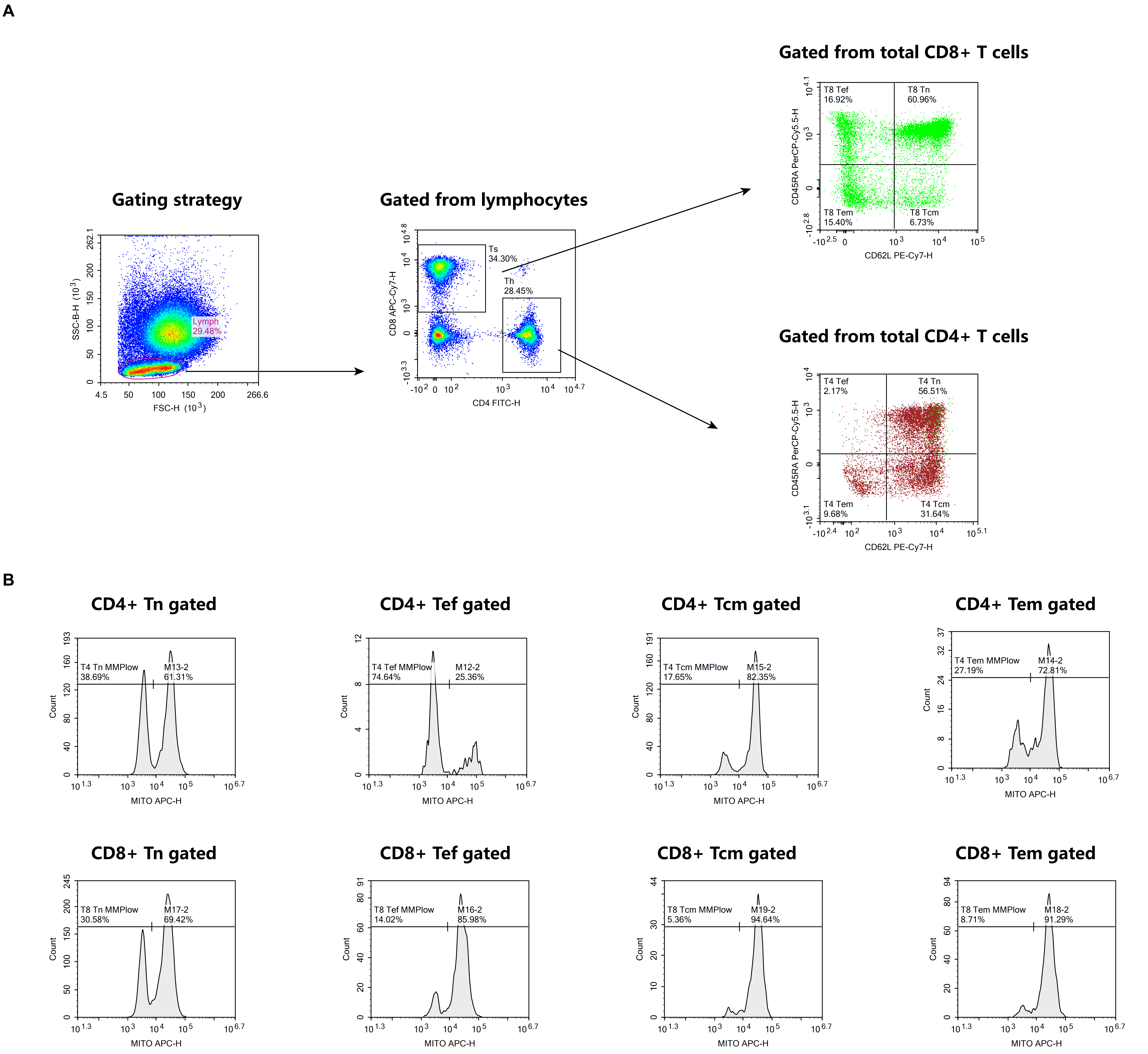

Supplementary Figure 2. Flow cytometry of CD4 and CD8 T lymphocyte subsets mitochondrial mass. **(A)** The gating strategies for lymphocyte, CD4+ T, CD8+ T, CD4+ Tn, CD4+ Tef, CD4+ Tcm, CD4+ Tem, CD8+ Tn, CD8+ Tef, CD8+ Tcm, CD8+ Tem cells using flow cytometry. **(B)** Representative flow cytometry plots showing the MFI of MitoDye in CD4+ Tn, CD4+ Tef, CD4+ Tcm, CD4+ Tem , CD8+ Tn, CD8+ Tef, CD8+ Tcm, CD8+ Tem cells subsets.
