# Supplementary material for: Mitochondrial mass and mitochondrial membrane potential of peripheral lymphocytes: promising biomarkers of systemic lupus erythematosus
Source: Front Mol Biosci. 2025 Jun 6;12:1585847. doi: 10.3389/fmolb.2025.1585847 (PMC12178850; doi:10.3389/fmolb.2025.1585847)
Supplement: Supplementary file 3 [file Image3.pdf]

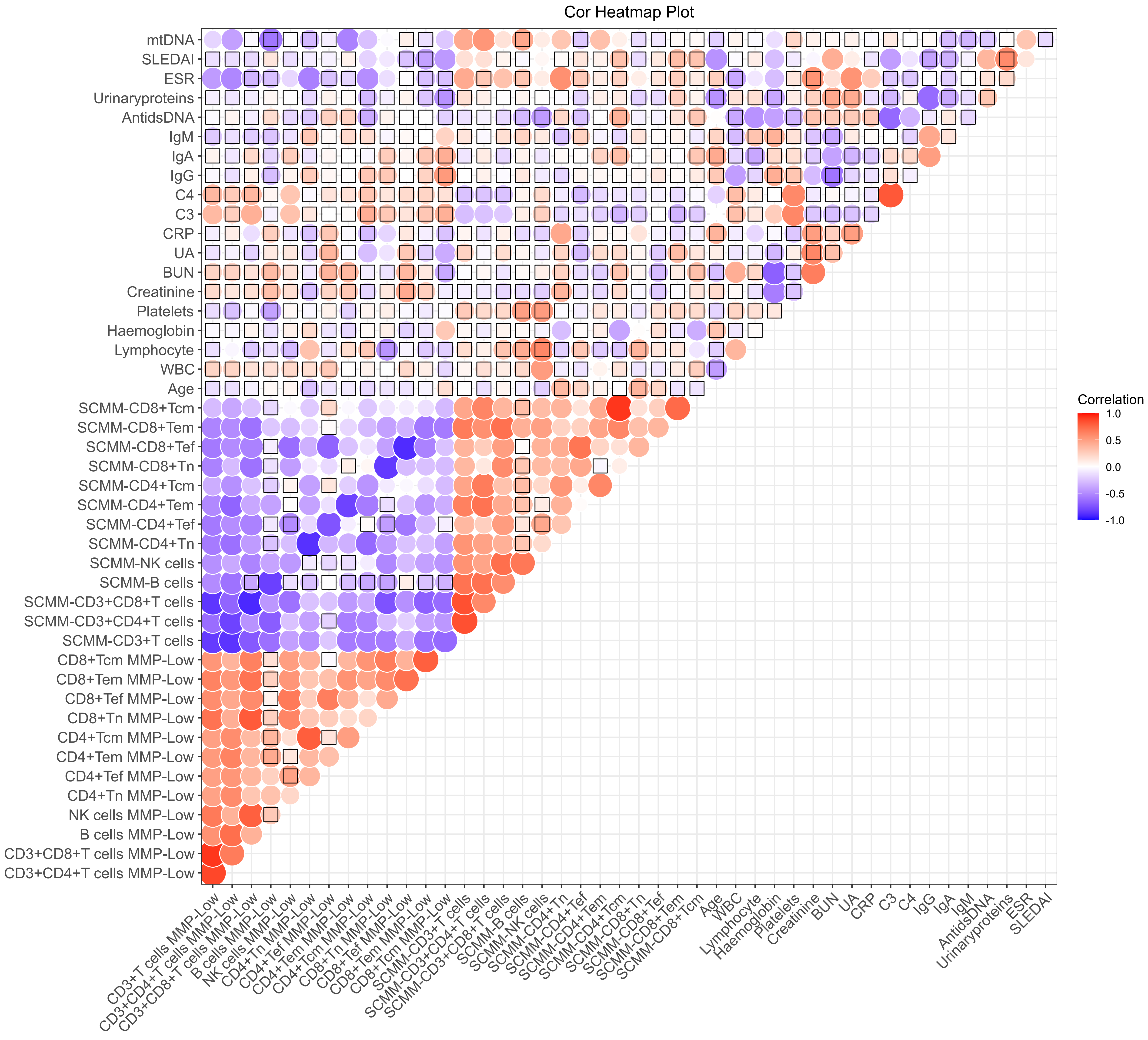

Supplementary Figure 3. Spearman correlation test was used to assess the possible relationship between the level of mitochondrial damage in immune cells and the value of laboratory examination and SLEDAI-2000
