# Supplementary material for: Mitochondrial mass and mitochondrial membrane potential of peripheral lymphocytes: promising biomarkers of systemic lupus erythematosus
Source: Front Mol Biosci. 2025 Jun 6;12:1585847. doi: 10.3389/fmolb.2025.1585847 (PMC12178850; doi:10.3389/fmolb.2025.1585847)
Supplement: Supplementary file 4 [file Image1.pdf]

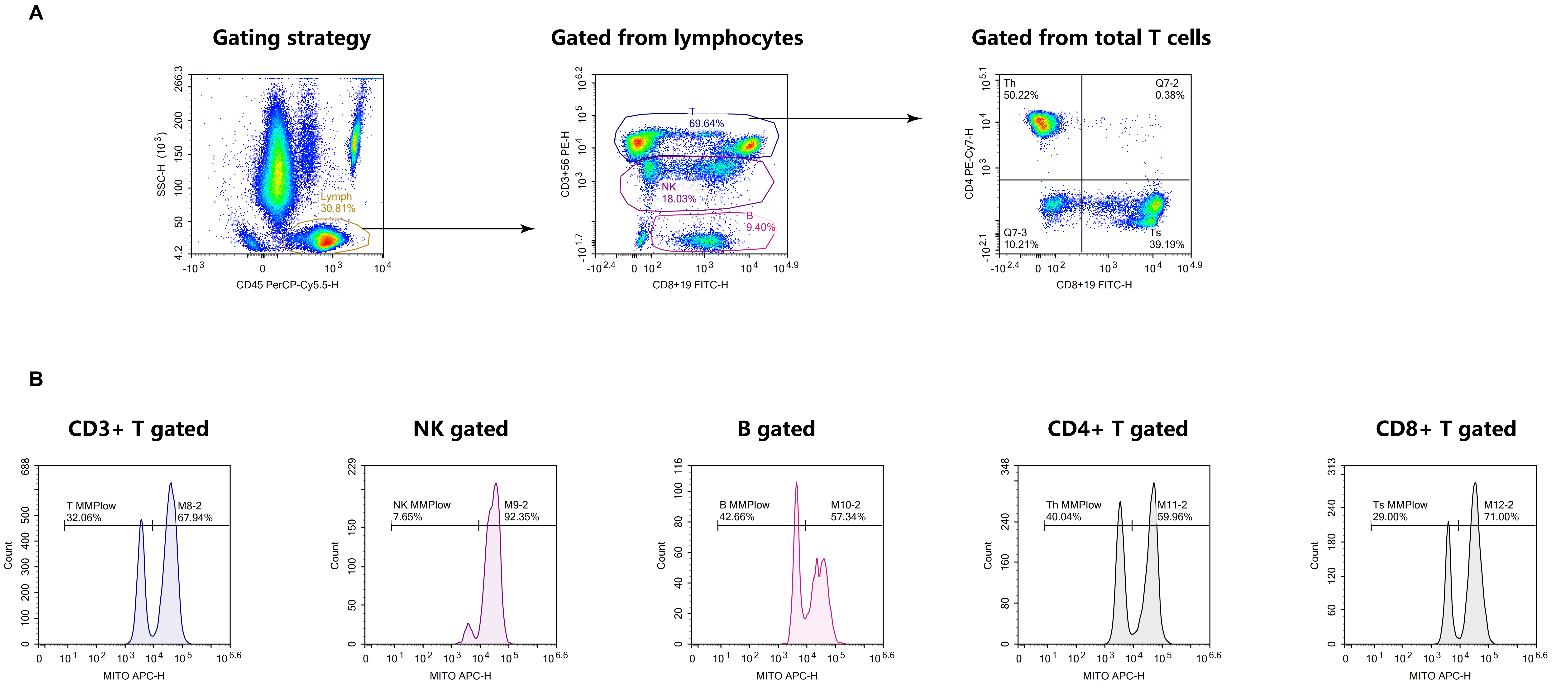

Supplementary Figure 1. Flow cytometry of T, B, NK lymphocyte mitochondrial mass. **(A)** The gating strategies for lymphocyte, CD3+ T, B, NK, CD4+ T, CD8+ T cells using flow cytometry. **(B)** Representative flow cytometry plots showing the MFI of MitoDye in CD3+ T, B, NK, CD4+ T, CD8+ T cells subsets.
